# Human papillomavirus 16 infection as a potential risk factor for prostate cancer: an adaptive meta-analysis

**DOI:** 10.4178/epih/e2015005

**Published:** 2015-02-11

**Authors:** Jong-Myon Bae

**Affiliations:** Department of Preventive Medicine, Jeju National University School of Medicine, Jeju, Korea

**Keywords:** Prostate neoplasms, Human papillomavirus 16, Risk factor, Meta-analysis, Oncogenic viruses

## Abstract

**OBJECTIVES:**

Although an expert review published in 2013 concluded that an association between human papillomavirus (HPV) infection and prostate cancer (PCa) risk had not yet been firmly established, a 2011 systematic review of 14 articles revealed an increased prevalence of HPV-16 DNA in PCa tissues. Another meta-analysis of the related articles is needed to evaluate the potential link between HPV infection and PCa risk.

**METHODS:**

A snowballing search strategy was applied to the previously cited articles in the above-mentioned expert review and systematic review. Additional articles selected for this meta-analysis should fulfill all following inclusion criteria: (a) evaluation of detected HPV-16 DNA in tissue samples and the PCa risk and (b) report of the HPV-16 prevalence in both cancer and control tissues. Estimated summary odds ratios (sOR) with 95% confidence intervals (CI) were calculated using fixed effect or random-effect models.

**RESULTS:**

Hand searching identified 16 new articles. The sOR of the total 30 articles indicated a significant HPV-16 infection-related increase in the PCa risk (sOR, 1.851; 95% CI, 1.353 to 2.532, I^2^=37.82%).

**CONCLUSIONS:**

These facts provide additional supportive evidence for a causal role of HPV-16 infection in prostate carcinogenesis. As the PCa incidence rates have increased rapidly in Asian countries, including Korea, during the last several decades, further studies of HPV-related PCa carcinogenesis may be necessary.

## INTRODUCTION

**Figure 1.**
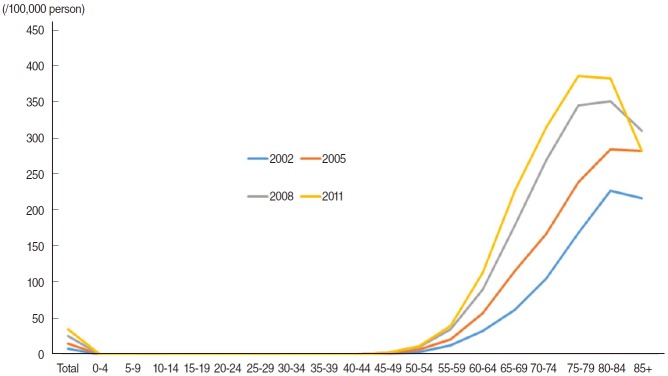
Age-adjusted incidences of prostate cancer in Korean men in 2002, 2005, 2008, and 2011 according to age group.

Prostate cancer (PCa) represents a major type of cancer in western societies [1], and recently the incidence and mortality rates have been increasing in Asian societies [2-5]. As shown in Figure 1, the PCa incidence rate in Korea has increased continuously during the last decade [6], a finding that has been attributed to an increasingly westernized diet [3-5]. On the other hand, the age group with the highest PCa incidence rate has changed from the 80 to 84 years group to the 75 to 79 years group (Figure 1). This pattern can be interpreted as the result of PCa overdiagnosis [7,8].

Non-modifiable factors such as old age, race, or family history are commonly accepted PCa risk factors [9,10]. However, epidemiological studies for factors with the potential for preventable intervention, including smoking [11], drinking [12], exercise [13], and diet [14] showed as controversial [15]. Recently, despite a widely publicized systematic review in which a diabetes history was associated with a reduced PCa incidence [16,17], an opposing report stated that diabetes increased the incidence of PCa in Asian populations [18]; therefore, it is necessary to carefully interpret results [19].

On the other hand, chronic recurrent inflammation is known to cause PCa, and theories regarding the etiological mechanism have been established [20-22]. In this context, prostatitis [23] and venereal disease infectivity [24] have been suggested as risk factors for PCa. Given the chance that subject reporting bias could be introduced when measuring the infectivity of venereal disease, however, it was noted that the study results might have been inconsistent [25]. On the other hand, human papillomavirus (HPV) infection has been strongly suggested as a risk factor for PCa [26-30]. This suggestion is based on the identification of HPV as a causative risk factor for urogenital system cancers such as cervical cancer and penile cancer [31,32] and the finding that HPV infection as a type of sexually transmitted infection [33,34], causes chronic recurrent inflammation [26]. Moreover, one suggested hypothesis stated that the incidence of breast cancer, which is known to increase along with changes toward a more western lifestyle, is also caused by HPV infection [35,36].

Among the studies conducted to investigate whether HPV infection is a risk factor for PCa, Lin et al. [27] published a systematic review paper (SRP) in 2011 in which a meta-analysis had been applied. The authors concluded that although there was no general association, statistical significance was observed when the analysis was limited to HPV DNA detection of type 16 infection in PCa tissues; therefore, they concluded that the causality remained doubtful. However, a 2013 study by Hrbacek et al. [25] investigated the likelihood that various infections, including HPV, might be risk factors for PCa during the previous three decades; this study concluded, however, that there was no evidence to support an association. However, the EXP study design was an expert review (EXP) without a meta-analysis. As such, although the SRP and EXP reached inconsistent conclusions, the most recent papers cited in the SRP and EXP to demonstrate statistical significance in the detection of HPV type 16 DNA in tissues were published in 2010 and 2011, respectively. Now, in January 2015, it is necessary to perform another meta-analysis including papers newly published since the previous reviews. Thereby, the purpose of the present study was to investigate whether HPV type 16 infection is a risk factor for PCa by performing a meta-analysis using adaptive papers related to HPV type 16 DNA detection in PCa tissues.

## MATERIALS AND METHODS

### Searches and selection of related papers

As the SRP and EXP papers had already been published, it is necessary to set the same selection criteria and fully apply the list of selected subjects to perform another meta-analysis. Thereby, the EXP subject criteria were first maximally reflected for the present study, and the papers were narrowed to case-control studies in which the HPV type 16-related DNA prevalence levels were compared between PCa and control tissues according to the purpose of the study. Finally, papers that did not provide information about type 16 or the number of HPV DNA detection positive tissues were excluded.

In addition, to ensure a more efficient literature search and maximize the use of the subject lists in the SRP and EXP, the snowballing search strategy was applied to manually search for the referred literature in each paper [37]. Therefore, the subjects of the present study were divided into three groups (A, B, and C groups). First, the A group included 14 papers listed as subjects in the SRP (published in 2011) [38-51]. Second, the B group comprised papers that satisfied the selection criteria but had been absent from the A group; these papers were identified from a mutual comparison with the list presented in the EXP (published in 2013). Third, the C group contained papers that satisfied the selection criteria from the list of papers related to each paper in the A and B groups. As papers involving the same study hypothesis are often mutually referent, the search list was created using the list of “Related citations” for each paper as provided by PubMed (National Library of Medicine, USA); subsequently, a manual hand search was performed, followed by the selection of subjects that satisfied the selection criteria [52].

### Statistical analysis

The total numbers of cancer and control tissues and samples with extracted HPV type 16 DNA were identified in the selected papers. Heterogeneity was evaluated using I^2^ values (%) [53], and a meta-analysis was performed to calculate the summary odds ratios (sOR) and 95% confidence intervals (CI) according to the fixed effect model and random effect model, using the OR value of each paper. Furthermore, in consideration of the improved accuracy of HPV DNA detection methods in more recent studies [36,54], a subgroup analysis was performed after the papers were divided depending on the publication before and after the year 2000. Finally, to evaluate any publication bias, the funnel plot symmetry was tested and the Egger regression was applied. The threshold of statistical significance was set at 5%, and the STATA/SE version 13.0 (StataCorp., College Station, TX, USA) was used for the meta-analysis and creation of the two plots.

## RESULTS

The numbers of detected positive cases in the 30 selected subject papers are presented in Table 1. Among the 14 papers selected from the SRP [38-51], the number of detected DNA-positive cases reported in the paper by Terris and Peehl [44] was different from that presented in the SRP, and accordingly this number was corrected. After excluding nine papers [55-63] with no information about HPV type 16 among the 17 papers present in the EXP list but absent from the A group, eight papers [64-71] were added to the B group. Each of these 22 papers and the literature cited in EXP and SRP were manually searched using the list of “Related citations” provided by PubMed; the selection criteria were applied to this search, resulting in the addition of eight new related papers to the C group [26,72-78]. Interestingly, one paper published before 2000 was included in the C group [72].

**Figure 2.**
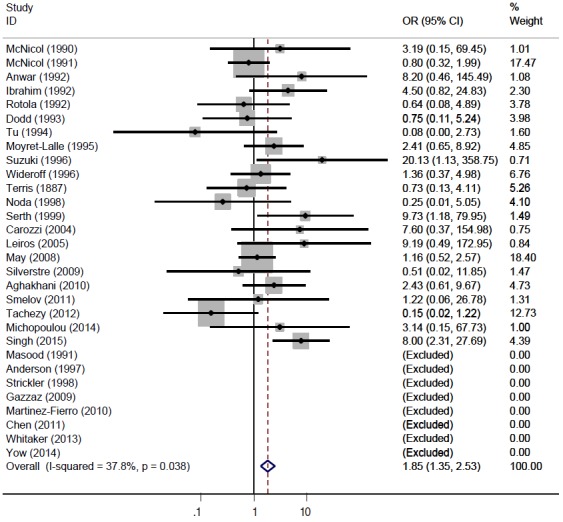
Forest plot of all selected articles (n = 30). OR, odds ratio; CI, confidence interval.

**Figure 3.**
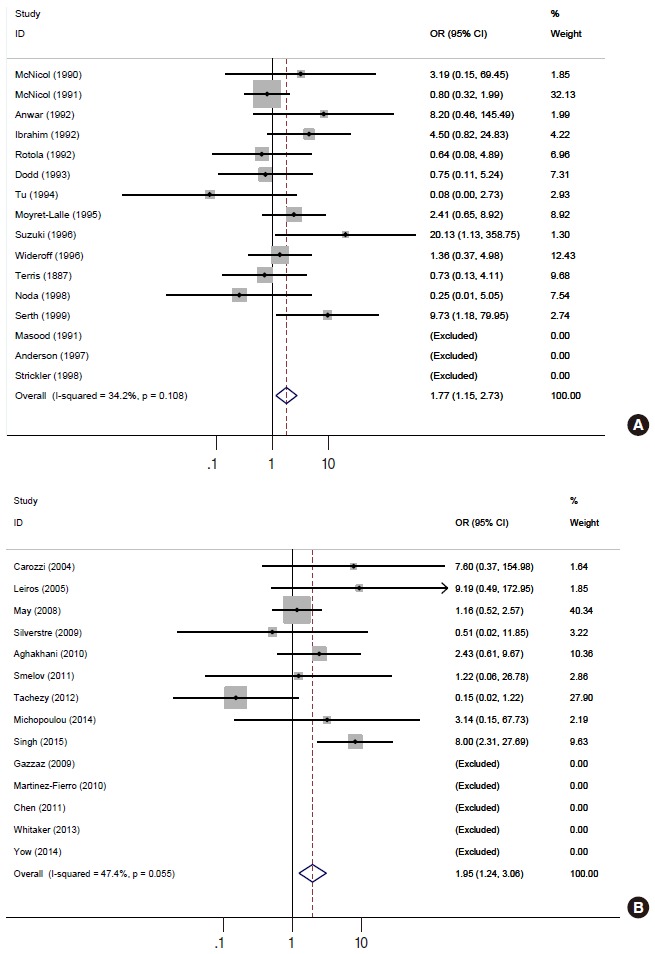
Forest plots of subgroup analyses according to publication year before (n=16, A) and after 2000 (n=14, B). OR, odds ratio; CI, confidence interval.

**Figure 4.**
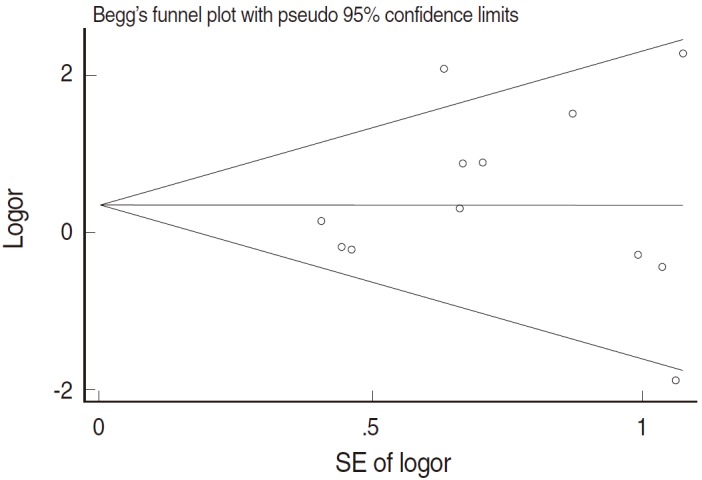
Funnel plot with Egger’s test (t=0.64, p=0.537). SE, standard error; logor, log odds ratio.

Figure 2 and Table 2 present the results of the meta-analysis of a total of 30 papers. As eight papers featured weights of 0% because of the absence of DNA detection in both cancer and control tissues, the sOR of the fixed effect model in the A group alone was 1.669 (95% CI, 1.134 to 2.456); this was higher than the sOR (1.54) presented in the SRP. This result was obtained after correcting the values obtained from the paper by Terris and Peehl [44]. When the B and C groups were added to this analysis, although the I^2^ value increased from 27.78% to 37.2%, the sOR of the fixed effect model increased to 1.851 (95% CI, 1.353 to 2.532); the sOR of the random effect model also achieved statistical significance (sOR, 1.719; 95% CI, 1.037 to 2.848). When a subgroup analysis was performed according to publication year in consideration of variations in the test method accuracy over time, the sOR of the publications after the year 2000 was higher than that of publications before the year 2000, and no change was observed in the statistical significance of the sOR calculated using the fixed effect model (Figure 3). On the other hand, a funnel plot (Figure 4) used to test publication errors was symmetrical, and the Egger regression results also indicated a low likelihood of error (p=0.537).

## DISCUSSION

The results of our adaptive meta-analysis, which added 16 papers to the list analyzed in the previously published SRP, additionally support the hypothesis that HPV-16 infectivity, as determined by DNA detection in tissues, increases the risk of PCa incidence. This introduces a theoretical background on which HPV infectivity must be considered as a risk factor for PCa despite of the controversial conclusions of studies conducted in the past three decades, as shown in the EXP, and indicates the necessity of further studies.

As such, it is necessary to actively conduct research on HPV infectivity as a risk factor for PCa because the findings could lead to the establishment of chemopreventive and immunophylactic strategies to prevent the incidence of related cancers [33]. First, a chemopreventive strategy involving the administration of an anti-inflammatory agent could be devised against chronic and recurrent inflammatory responses resulting from HPV infection [79-81]. This suggestion is supported by a paper published in 2012 [82] in which the administration of aspirin reduced the PCa mortality rate. Second, the findings of such research could lay a theoretical foundation for PCa prevention via HPV vaccination [83] and could provide the additional effect of reduced cervical cancer and breast cancer incidence rates among spouses [36].

The major limitation of the present study is the skip of an online search process required for systematic reviews [52]. However, the present study fully exploited the results of both wide and concentrated searches in the previously published SRP and EXP. In contrast, both time and manpower were expended upon manual searches for each cited paper in the references from both papers to ensure that papers published after SRP and EXP could be secured more efficiently. Using this process, an additional paper [72] that had been excluded despite satisfying the selection criteria was identified, and two other papers [44,49] were identified as present on the SRP list but absent from the EXP list. These findings demonstrate the limitations of a reference search strategy in combination with the existing online search. The snowballing search strategy used in the present study, which is the main search strategy used in narrative review and is difficult to establish [37], is expected to be used effectively for adaptive analyses of preexisting systematic review studies in the future.

Currently, active countermeasures are required for PCa care because infection is the major etiological agent of cancer worldwide [84-86]; additionally, PCa mostly occurs in older men and Korean society is aging rapidly [87]. Accordingly, it will be necessary to perform active epidemiological studies to reveal causal relationships between PCa and etiological events, particularly with respect to HPV infectivity [33]. HPV infection can be evaluated either in tissues via DNA detection methods or in sera via serological test methods; the limitations of each method have been well documented in the report by Hrbacek et al. [25]. Recently, the numbers of serological test-based studies have increased, but these are limited because seroconversion occurs in 50-60% of all HPV-infected people [88]. Review and reflection on these points are therefore recommended when planning a research proposal.

## Figures and Tables

**Table 1.** The subject articles (n = 30) in the adaptive meta-analysis for evaluating human papillomavirus 16 infection as a potential risk factor for prostate cancer

Group	Author	Year	Ref	Case_T	Case_P	Contrd_T	Contral_P
A	McNicol & Dodd	1991	38	27	14	61	35
A	Anwar et al.	1992	41	68	11	20	0
A	Ibrahim et al.	1992	40	24	6	29	2
A	Rotola et al.	1992	39	8	6	17	14
A	Tu et al.	1994	42	60	1	1	0
A	Moyret-Lalle et al.	1995	43	17	9	22	7
A	Terris & Peehl	1997	44	53	2	78	4
A	Noda et al.	1998	45	38	0	71	3
A	Serth et al.	1999	46	47	10	37	1
A	Carozzi et al.	2004	47	26	3	25	0
A	Leiros et al.	2005	48	41	5	30	0
A	May et al.	2008	49	50	10	163	29
A	Silverstre et al.	2009	50	65	2	6	0
A	Aghakhani et al.	2011	51	104	7	104	3
B	Masood et al.	1991	64	20	0	20	0
B	Dodd et al.	1993	65	7	3	10	5
B	Suzuki et al.	1996	66	51	8	51	0
B	Wideroff et al.	1996	67	56	7	42	4
B	Anderson et al.	1997	68	14	0	10	0
B	Strickler et al.	1998	69	63	0	61	0
B	Gazzaz & Mosli	2009	70	6	0	50	0
B	Martinez-Fierro et al.	2010	71	55	0	75	0
C	McNicol & Dodd	1990	72	4	4	20	15
C	Chen et al.	2011	73	51	0	11	0
C	Smelov et al.	2011	74	61	2	14	0
C	Tachezy et al.	2012	75	51	1	95	11
C	Whitaker et al.	2013	26	10	0	20	0
C	Michopoulou et al.	2014	76	50	2	30	0
C	Yow et al.	2014	77	115	0	51	0
C	Singh et al.	2015	78	95	30	55	3

A, selected articles from reference [27]; B, selected articles from a comparison of subjects in reference [27] and reference [25]; C, selected articles via manual searching; Ref, reference number; Case_T, total number in the case group; Case_P, number of positive cases; Control_T, total number in the control group; Control_P, number of positive controls.

**Table 2.** Summary odds ratio (sORs) and 95% confidence intervals (CIs) from a meta-analysis with subgroup analysis for evaluating human papillomavirus 16 infection as a potential risk factor for prostate cancer

Group and subgroup	n (df)	I^2^ statistic (%)	sOR (Fixed) [95% CI]	sOR (Random) [95% CI]		
A	14 (13)	27.78	1.669 [1.134, 2.456]	1.574 [0.906, 2.735]		
A+B	22 (16)	27.43	1.761 [1.235, 2.509]	1.600 [0.968, 2.643]		
A+B+C	30 (21)	37.82	1.851 [1.353, 2.532]	1.719 [1.037, 2.848]		
Publication before 2000	16 (12)	34.24	1.772 [1.150, 2.729]	1.565 [0.820, 2.987]		
Publication after 2000	14 (8)	47.41	1.946 [1.236, 3.064]	1.961 [0.812, 4.733]		

A, selected articles from reference [27]; B, selected articles from a comparison of subjects in reference [27] and reference [25]; C, selected articles via manual searching. df, degree of freedom; Fixed, fixed effect model; Random, random effect model.
