# Supplementary material for: Human papillomavirus 16 infection as a potential risk factor for prostate cancer: an adaptive meta-analysis
Source: Epidemiol Health. 2015 Feb 11;37:e2015005. doi: 10.4178/epih/e2015005 (PMC4371392; doi:10.4178/epih/e2015005)
Supplement: Supplementary file 1 [file epih-37-e2015005-supplementary.pdf]

<Reviews>

전립선암 위험인자로서의 인간 유두종바이러스 16형 감염

# : 개작 메타분석

Human Papillomavirus 16 Infection as a Potential Risk factor of Prostate Cancer: Adaptive Meta-Analysis

Abstract

Objectives: While an expert review published in 2013 concluded that the association between human papilloma virus (HPV) infection and prostate cancer (PCa) risk has not established firmly yet, a systematic review published in 2011 revealed higher HPV-16 DNA prevalence in PCa tissue using 14 articles. To evaluate the potential link between HPV infection and PCa risk, another meta-analysis with adapting the related articles would be needed.

Methods: Based on the articles selected by the expert review and systematic review each, the cited articles were searched using snow-balling strategy. The selected articles should fulfill all the following inclusion criteria: (a) articles evaluating the HPV-16 DNA detection in tissue sample and PCa risk, and (b) showing the HPV-16 prevalence in both cancer and control tissues. Estimate of summary odds ratio (sOR) with 95% confidence intervals (CI) were calculated using the fixed effect or random-effect models.

Results: Hand searching let 16 new articles obtained. The sOR of total 30 articles indicated significant increase of PCa risk related with HPV-16 infection (sOR = 1.851, 95% CI: 1.353 – 2.532, I^2 = 37.82%).

Conclusions: These facts provide additionally supportive evidence for causal role of HPV-16 in prostate carcinogenesis. As incidence rates of PCa during the last several

decades have been rapidly increasing in Asia countries including Korea, further studies for carcinogenesis of HPV infection in PCa may be necessary.

Key words: Prostate neoplasms, Human papillomavirus 16, Risk factor, meta-analysis, Oncogenic viruses

Introduction

전립선암은 서구 사회의 주요 암에 속하며 [1], 최근 아시아권에서도 발생률 및 사망률이 증가하고 있다[2-5]. 한국도 Fig. 1에서 보듯이 지난 10년간 발생률이 꾸준히 증가하고 있는데[6], 이에 대하여는 서구화된 식습관 변천으로 해석하고 있다[3-5]. 그런데 Fig 1.에서 발생률이 최고가 되는 연령군이 80-84세 군에서 75-79세 군으로 낮아지는 양상을 보이고 있는 사실에서, 전립선암의 과다 검진이 이루어진 결과로도 해석을 할 수 있다[7,8].

전립선암의 위험인자로 이견이 없는 것은 고령, 인종, 가족력 같은 교정이 불가능한 (non-modifiable) 인자들이다[9,10]. 반면 예방 개입 (preventable intervention)이 가능한 흡연[11], 음주[12], 운동[13], 식이[14] 등에서 여전히 상반된 역학 결과가 발표되고 있다[15]. 최근 당뇨력이 전립선암 발생을 감소시킨다는 체계적 고찰 (systematic review) 결과가 보고되어 관심을 끌었지만[16,17], 아시아인에 있어서는 오히려 발생을 높인다는 보고[18]도 있어 결과 해석에 주의를 요한다[19].

한편, 만성 재발성 염증 (chronic recurrent inflammation)이 전립선암 발생을 야기하며, 그 기전에 관한 이론들이 정립되고 있다[20-22]. 이런 배경 하에, 전립선염[23]과 성병감염력[24]이 전립선암의 위험인자란 주장이 있다. 그런데 성병 감염력 여부 측정에 있어 대상자의 보고 오류 (reporting bias)가 개입될 소지가 있어 연구결과가 일관성이 없다는 지적[25] 속에, 인간 유두종 바이러스 (Human Papillomavirus, 이하 HPV) 감염이 전립선암의 위험인자로 적극 제기 되어 왔다[26-30]. 그 배경에는 HPV가 자궁경부암, 음경암 등의 비뇨생식계 암을 일으키는 위험인자로 확인되었고[31,32], HPV 감염은 성 매개 감염의 일종이며 [33,34], 재발 및 만성 염증을 일으키기 때문이다[26]. 더군다나 서구형 생활습관 변화에 따

라 증가한다는 유방암도 HPV 감염에 의한 발생 가설이 제기되고 있다[35,36].

HPV 감염이 전립선암의 위험인자인가를 규명한 연구결과들에 있어 메타분석을 적용한 체계적 고찰 (systematic reviews)로는 2011년도에 발표된 Lin et al. [27] 논문이 있다(이하 SRP). 저자는 전반적으로 관련성이 없다는 결론을 내고 있지만, 16형 감염에 대한 전립선암 조직내 HPV DNA 검출여부로 한정하였을 때 통계적 유의성을 보인 점에서 인과성에 대한 의문을 여전히 가져야 한다고 결론내었다. 그러나 2013년도에 발표된 Hrbacek et al. [25] 논문(이하 EXP)에서는 지난 30년간 전립선암의 위험요인으로 HPV를 포함한 각종 감염의 가능성을 연구를 했지만 관련성에 대한 근거를 확보하지 못하였다고 결론내렸다. 그런데 이 EXP의 연구설계는 메타분석 (meta-analysis)을 적용하지 않은 전문가 고찰 (Expert review)이다. 이렇듯 SRP, EXP 두 문헌고찰 논문에서도 결론이 일치되지 않는 가운데, SRP에서 통계적 유의성을 보인 부분 – 조직에서의 HPV 16 DNA 검출-에 대한 최신 발간 연도가 각각 2010, 2011년도이었다. 현재 2015년 1월을 감안할 때, 그 이후 새로이 발표된 관련 논문을 추가로 확보하여 메타분석을 다시 해 볼 필요가 있겠다. 따라서 본 연구의 목적은 전립선암 조직내 HPV 16형 DNA 검출과 관련한 논문들을 추가 (adaptive)한 다음 메타분석을 재 시행하여 HPV 16형 감염이 전립선암 위험요인인가를 규명하는 것이다.

Subjects & Methods

가. 관련한 논문 검색 및 선정

이미 SRP와 EXP 논문이 발표된 상황에서, 메타분석을 재시행하기 위해서는 선정기준을 같게 하고 선정된 대상 목록을 최대한 활용할 필요가 있다. 이에, 본 연구 대상 기준은 우선적으로 EXP의 것을 최대한 반영하면서 연구목적에 맞추어, 전립선암 조직과 그 대조 조직간에 HPV 16관련 DNA 유병수준을 비교한 환자-대조군 연구로 좁혔다. 그리고 내용상 HPV DNA 검출에 있어 16형에 관한 정보가 없거나, 검출 양성자 수를 알 수 없는 경우는 제외하였다.

또한 문헌 검색에서 SRP와 EXP 대상 목록을 최대한 활용하면서, 보다 효율적인 탐색을 위하여 각 문헌을 중심으로 한 인용된 문헌들을 손으로 직접 찾는 snow-balling 검색전략을 구사하였다[37]. 따라서 본 연구 대상은 크게 3 군으로 나누어진다. 첫째, 2011년도에 발표된 SRP의 분석대상이 된 14개의 논문들로, 이를 A 군이라 한다[38-51]. 둘째, 2013에 발표된 EXP에서 제시한 목록과 상호 비교하여, A군에 없지만 선정기준에 맞는 논문들로 이를 B군이라 한다. 셋째, A, B 두 군에 속한 각각 논문과 관련한 논문 목록들 중에서 선정기준에 맞는 논문들로, 이를 C군이라 한다. 이는 연구가설이 같은 논문들은 상호 인용을 한다는 점을 감안하여 PubMed (National Library of Medicine, US) 에서 각 논문마다 'Related citations' 목록을 제공하고 있는 것을 활용하여 검색할 목록을 만든 다음 일일이 손으로 검색하고 (hand search) 선정기준에 맞는 대상을 선정하였다[52].

나. 통계분석

선정된 논문에 있어서 암조직과 대조조직 간의 총 수와 HPV 16 DNA 추출 양성자 수치를 알아내었다. 이질성 여부를 I^2 값(%)으로 평가하였으며[53], 논문별 OR값을 이용하여 고정효과모형 (fixed effect model)과 랜덤효과모형 (random effect model)에 따른 summary odds ratio (이하 총OR)과 이의 95% 신뢰구간 (confidence intervals, 이하 CI)을 구하는 메타분석을 시행하였다. 한편, 최신으로 올수록 HPV DNA 검출법의 정확도가 향상된다는 사실[36,54]을 고려하여, 2000년도를 이전과 이후로 나눈 하부군 분석 (subgroup analysis)를 시행하였다. 마지막으로 출판오류 여부를 평가하기 위하여 funnel plot의 대칭성 여부를 확인하고 Egger 회귀법을 적용하였다. 통계적 유의수준을 5%로 정했으며, 메타분석과 두 개의 plot 작성을 위해 StataSE 13 통계프로그램 (STATA Co., Texas, US)을 사용하였다.

Results

Table 1은 최종 선정된 30편의 대상 논문에 있어 암조직과 대조조직의 총 명수와 검출양성자 수를 제시한 것이다. SRP에서 선정된 총 14개의 논문[38-51] 중 Terris & Peehl 논문[44]에서 DNA 검출 양성자 숫자가 SRP의 제시내용과 다름을 확인하고 수치를 수정하였다. 목록 (A 군)에는 없으나 EXP 목록에 있는 17 개 논문 중, 16형에 대한 정보가 없는 9 개 논문[55-63]을 제외한 8 편의 논문[64-71]이 B군으로 추가되었다. 이들 22편과 EXP, SRP 문헌 각각을 PubMed 가 제공하는 'cited citations' 목록을 이용하여 손으로 검색하고 선정기준을 적용한 결과, 8편의 관련 논문을 C군으로 새로이 확보하였다[26, 72-78]. 특이한 것은 C군 중에서 2000년 이전에 발표된 논문이 한 편 있다 [72].

Fig. 2과 Table 2는 총 30편에 대한 메타분석 결과를 제시한 것이다. 암조직과 대조조직 모두 DNA 검출이 없어 가중치가 0%인 8편을 감안한 가운데, A 군만을 이용한 고정효과 모형의 sOR은 1.669 (95% CI: 1.134, 2.456)으로 SRP 제시한 sOR = 1.54 보다 증가하였다. 이는 Terris & Peehl 논문[44]의 관련 수치를 수정한 결과이다. 여기에 B군과 C군을 더했을 때, I^2값이 27.78%에서 37.2%로 증가하지만 고정효과 모형의 sOR 은 1.851 (95% CI: 1.353-2.532)로 더 높아졌을 뿐만 아니라, 랜덤효과모형에 따른 sOR에서도 통계적 유의성이 확보되었다 (sOR =1.719; 95% CI: 1.037-2.848). 연도별로 검사기법의 정확성 변동을 염두에 두고 출판연도별로 하부군 분석을 했을 때, 2000년 이전에 비하여 2000년 이후인 군의 sOR이 증가하였으며, 고정효과 모형으로 구한 sOR의 통계적 유의성은 변동이 없었다 (Fig 3). 한편 출판오류를 확인하기 위한 funnel plot (Fig. 4)에서 대칭성을 보였고, Egger 회귀법 결과도 오류 가능성이 낮은 것으로 나왔다 (P=0.537).

Discussion

기존에 발표된 SRP에 16편을 추가한 메타분석 결과는 조직내 DNA 검출여부에 따른 HPV-16 감염력이 전립선암의 발생 위험을 증가시킨다는 추가 근거가 된다. 이는 EXP의 지난 30년간의 연구성과에 대한 회의적인 결론에도 불구하고, HPV 감염력이 전립선암의 위험요인으로 여전히 의심할 필요가 있으며, 이에 대한 추가적 연구가 이루어져야 한다는 이론적 배경이 된다.

이처럼 HPV 감염력이 전립선암의 위험인자임을 규명하는 연구를 적극 수행해야 하는 이유에는 해당 암을 예방하기 위한 화학예방 및 면역예방 전략을 수립할 수 있기 때문이다[33]. 첫째로 HPV 감염에 의한 만성, 재발성 염증반응에 있어 항염증제를 투여하는 화학예방 전략을 강구할 수 있겠다[79-81]. 2012년 발표된 논문[82]에서 아스피린투여로 전립선암에 의한 사망을 줄인다는 보고는 이를 지지하는 근거이다. 둘째로 HPV 백신 접종을 통해 전립선암을 예방할 수 있다는 이론적 근거를 마련해 주며[83], 추가로 배우자의 자궁경부암, 유방암 발생 감소의 효과도 같이 가져다 줄 수 있기 때문이다[36].

본 연구의 주요 한계점은 체계적 고찰에서 반드시 요구되는 검색식을 적용한 온라인 검색 작업[52]을 거치지 않은 것이다. 그렇지만 본 연구는 기존에 발표된 SRP와 EXP 논문에서 이미 광범위하고 집중적인 검색작업을 수행했던 결과물을 최대한 활용하였다. 오히려 두 논문에서 선정한 문헌 각각에 대한 인용 논문들을 손으로 찾는 작업에 시간과 인력을 투입하여 두 논문 발표 이후의 논문들을 보다 효율적으로 확보할 수 있었다. 이 과정에서 논문이 제시한 선정 기준에 맞으나 제외되었던 한 개의 논문[72]을 추가로 알아내었을 뿐만 아니라, SRP 목록에는 있으나 EXP 목록에 없는 논문도 두 편 [44,49] 있었다. 이는 기존의 온라인 검색에 이은 참고문헌 검색전략으로는 한계가 있음을 보여준다. 검색전략을 세우기 어려

운 질적 연구 (narrative review)의 주된 검색전략인 snow-balling [37]을 이용한 본 연구의 검색 전략은 향후 기존의 체계적 고찰 연구의 추가 분석에 효과적으로 활용할 수 있을 것으로 기대한다.

전세계적으로 감염이 암 발생의 주요 인자이면서[84-86], 전립선암의 발생이 주로 고령 남성에서 이루어진다는 점과, 우리사회가 급속한 고령화를 거치고 있다는 점에서 전립선암 관리에 대한 적극적인 대응이 요구되는 시점이다 [87]. 이를 위해서는 발생요인들로 특히 HPV 감염력에 대한 인과관계를 규명하는 역학연구를 적극 수행해야 할 것이다 [33]. HPV 감염 여부는 조직내 DNA를 검출하는 법과, 혈청학 (serology)적 검사법이 있으며, 그 각각의 한계점을 Hrbacek et al. [25]의 문헌에서 잘 제시하고 있다. 최근 혈청학 검사법에 의한 연구가 증가하고 있으나, HPV 감염자의 50~60%에서 혈청학적 전환 (seroconvert)이 일어난다는 점에서 한계를 가진다 [39062]. 연구계획서 수립단계에서 이런 점들을 잘 검토하고 반영하기를 권한다.

감사의 글

이 논문은 2014학년도 제주대학교 학술진흥연구비 지원사업에 의하여 연구되었음

References

1. Ferlay J, Parkin DM, Steliarova-Foucher E. Estimates of cancer incidence and mortality in Europe in 2008. Eur J Cancer 2010;46:765-781.

2. Cullen J, Elsamanoudi S, Brassell SA, Chen Y, Colombo M, Srivastava A, et al. The burden of prostate cancer in Asian nations. J Carcinog 2012;11:7.

3. Zhu Y, Wang HK, Qu YY, Ye DW. Prostate cancer in East Asia: evolving trend over the last decade. Asian J Androl 2015;17:48-57.

4. Namiki M, Akaza H, Lee SE, Song JM, Umbas R, Zhou L, et al. Prostate Cancer Working Group report. Jpn J Clin Oncol 2010;40 Suppl 1:i70-i75.

5. Zhang J, Dhakal IB, Zhao Z, Li L. Trends in mortality from cancers of the breast, colon, prostate, esophagus, and stomach in East Asia: role of nutrition transition. Eur J Cancer Prev 2012;21:480-489.

6. Jung KW, Won YJ, Kong HJ, Oh CM, Lee DH, Lee JS. Cancer statistics in Korea: incidence, mortality, survival and prevalence in 2011. Cancer Res Treat 2014;46:109-123.

7. Hilton WM, Padalecki SS, Ankerst DP, Leach RJ, Thompson IM. Temporal changes in the clinical approach to diagnosing prostate cancer. J Natl Cancer Inst Monogr 2012;2012:162-168.

8. Sandhu GS, Andriole GL. Overdiagnosis of prostate cancer. J Natl Cancer Inst Monogr 2012;2012:146-151.

9. Shavers VL, Underwood W, Moser RP. Race/ethnicity and the perception of the risk of developing prostate cancer. Am J Prev Med 2009;37:64-67.

10. Dong HV, Lee AH, Nga NH, Quang N, Chuyen VL, Binns CW. Epidemiology and prevention of prostate cancer in Vietnam. Asian Pac J Cancer Prev 2014;15:9747-9751.

11. Bae JM, Li ZM, Shin MH, Kim DH, Lee MS, Ahn YO. Cigarette smoking and prostate cancer risk: negative results of the Seoul Male Cancer Cohort Study. Asian Pac J Cancer Prev 2013;14:4667-4669.

12. Middleton Fillmore K, Chikritzhs T, Stockwell T, Bostrom A, Pascal R. Alcohol use and prostate cancer: a meta-analysis. Mol Nutr Food Res 2009;53:240-255.

13. Young-McCaughan S. Potential for prostate cancer prevention through physical activity. World J Urol 2012;30:167-179.

14. Gathirua-Mwangi WG, Zhang J. Dietary factors and risk for advanced prostate cancer. Eur J Cancer Prev 2014;23:96-109.

15. Wolk A. Diet, lifestyle and risk of prostate cancer. Acta Oncol 2005;44:277-281.

16. Xu H, Mao SH, Ding GX, Ding Q, Jiang HW. Diabetes mellitus reduces prostate cancer risk - no function of age at diagnosis or duration of disease. Asian Pac J Cancer Prev 2013;14:441-447.

17. Bansal D, Bhansali A, Kapil G, Undela K, Tiwari P. Type 2 diabetes and risk of prostate cancer: a meta-analysis of observational studies. Prostate Cancer Prostatic Dis 2013;16:151-158.

18. Long XJ, Lin S, Sun YN, Zheng ZF. Diabetes mellitus and prostate cancer risk in Asian countries: a meta-analysis. Asian Pac J Cancer Prev 2012;13:4097-4100.

19. Zhang F, Yang Y, Skrip L, Hu D, Wang Y, Wong C, et al. Diabetes mellitus and risk of prostate cancer: an updated meta-analysis based on 12 case-control and 25 cohort studies. Acta Diabetol 2012;49 Suppl 1:S235-S246.

20. Sfanos KS, Hempel HA, De Marzo AM. The role of inflammation in prostate cancer. Adv Exp Med Biol 2014;816:153-181.

21. De Marzo AM, Platz EA, Sutcliffe S, Xu J, Grönberg H, Drake CG, et al. Inflammation in prostate carcinogenesis. Nat Rev Cancer 2007;7:256-269.

22. Elkahwaji JE. The role of inflammatory mediators in the development of prostatic hyperplasia and prostate cancer. Res Rep Urol 2012;5:1-10.

23. Jiang J, Li J, Yunxia Z, Zhu H, Liu J, Pumill C. The role of prostatitis in prostate cancer: meta-analysis. PLoS One 2013;8:e85179.

24. Caini S, Gandini S, Dudas M, Bremer V, Severi E, Gherasim A. Sexually transmitted infections and prostate cancer risk: a systematic review and meta-analysis. Cancer Epidemiol 2014;38:329-338.

25. Hrbacek J, Urban M, Hamsikova E, Tachezy R, Heracek J. Thirty years of research on infection and prostate cancer: no conclusive evidence for a link. A systematic review. Urol Oncol 2013;31:951-965.

26. Whitaker NJ, Glenn WK, Sahrudin A, Orde MM, Delprado W, Lawson JS. Human papillomavirus and Epstein Barr virus in prostate cancer: Koilocytes indicate potential oncogenic influences of human papillomavirus in prostate cancer. Prostate 2013;73:236-241.

27. Lin Y, Mao Q, Zheng X, Yang K, Chen H, Zhou C, et al. Human papillomavirus 16 or 18 infection and prostate cancer risk: a meta-analysis. Ir J Med Sci 2011;180:497-503.

28. Al Moustafa AE. Involvement of human papillomavirus infections in prostate cancer progression. Med Hypotheses 2008;71:209-211.

29. Al-Maghrabi JA. The role of human papillomavirus infection in prostate cancer. Saudi Med J 2007;28:326-333.

30. Sutcliffe S, Viscidi RP, Till C, Goodman PJ, Hoque AM, Hsing AW, et al. Human papillomavirus types 16, 18, and 31 serostatus and prostate cancer risk in the Prostate Cancer Prevention

Trial. Cancer Epidemiol Biomarkers Prev 2010;19:614-618.

31. Korodi Z, Dillner J, Jellum E, Lumme S, Hallmans G, Thoresen S, et al. Human papillomavirus 16, 18, and 33 infections and risk of prostate cancer: a Nordic nested case-control study. Cancer Epidemiol Biomarkers Prev 2005;14:2952-2955.

32. Alibek K, Karatayeva N, Bekniyazov I. The role of infectious agents in urogenital cancers. Infect Agent Cancer 2012;7:35.

33. Ramezani A, Banifazl M, Eslamifar A, Aghakhani A. Association between human papillomavirus infection and risk of prostate cancer. Iran J Pathol 2011;6:3-7.

34. Rosenblatt KA, Carter JJ, Iwasaki LM, Galloway DA, Stanford JL. Serologic evidence of human papillomavirus 16 and 18 infections and risk of prostate cancer. Cancer Epidemiol Biomarkers Prev 2003;12:763-768.

35. Grover PL, Martin FL. The initiation of breast and prostate cancer. Carcinogenesis 2002;23:1095-1102.

36. Bae JM. Two hypotheses of dense breasts and viral infection for explaining incidence of breast cancer by age group in Korean women. Epidemiol Health 2014;36:e2014020.

37. Bae JM. Narrative reviews. Epidemiol Health 2014;36:e2014018.

38. McNicol PJ, Dodd JG. High prevalence of human papillomavirus in prostate tissues. J Urol 1991;145:850-853.

39. Rotola A, Monini P, Di Luca D, Savioli A, Simone R, Secchiero P, et al. Presence and physical state of HPV DNA in prostate and urinary-tract tissues. Int J Cancer 1992;52:359-365.

40. Ibrahim GK, Gravitt PE, Dittrich KL, Ibrahim SN, Melhus O, Anderson SM, et al. Detection of human papillomavirus in the prostate by polymerase chain reaction and in situ hybridization. J Urol 1992;148:1822-1826.

41. Anwar K, Nakakuki K, Shiraishi T, Naiki H, Yatani R, Inuzuka M. Presence of ras oncogene mutations and human papillomavirus DNA in human prostate carcinomas. Cancer Res 1992;52:5991-5996.

42. Tu H, Jacobs SC, Mergner WJ, Kyprianou N. Rare incidence of human papillomavirus types 16 and 18 in primary and metastatic human prostate cancer. Urology 1994;44:726-731.

43. Moyret-Lalle C, Marçais C, Jacquemier J, Moles JP, Daver A, Soret JY, et al. ras, p53 and HPV status in benign and malignant prostate tumors. Int J Cancer 1995;64:124-129.

44. Terris MK, Peehl DM. Human papillomavirus detection by polymerase chain reaction in benign and malignant prostate tissue is dependent on the primer set utilized. Urology 1997;50:150-156.

45. Noda T, Sasagawa T, Dong Y, Fuse H, Namiki M, Inoue M. Detection of human papillomavirus

(HPV) DNA in archival specimens of benign prostatic hyperplasia and prostatic cancer using a highly sensitive nested PCR method. Urol Res 1998;26:165-169.

46. Serth J, Panitz F, Paeslack U, Kuczyk MA, Jonas U. Increased levels of human papillomavirus type 16 DNA in a subset of prostate cancers. Cancer Res 1999;59:823-825.

47. Carozzi F, Lombardi FC, Zendron P, Confortini M, Sani C, Bisanzi S, et al. Association of human papillomavirus with prostate cancer: analysis of a consecutive series of prostate biopsies. Int J Biol Markers 2004;19:257-261.

48. Leiros GJ, Galliano SR, Sember ME, Kahn T, Schwarz E, Eiguchi K. Detection of human papillomavirus DNA and p53 codon 72 polymorphism in prostate carcinomas of patients from Argentina. BMC Urol 2005;5:15.

49. May M, Kalisch R, Hoschke B, Juretzek T, Wagenlehner F, Brookman-Amissah S, et al. Detection of papillomavirus DNA in the prostate: a virus with underestimated clinical relevance? Urologe A. 2008;47:846-852. (German)

50. Silvestre RV, Leal MF, Demachki S, Nahum MC, Bernardes JG, Rabenhorst SH, et al. Low frequency of human papillomavirus detection in prostate tissue from individuals from Northern Brazil. Mem Inst Oswaldo Cruz 2009;104:665-667.

51. Aghakhani A, Hamkar R, Parvin M, Ghavami N, Nadri M, Pakfetrat A, et al. The role of human papillomavirus infection in prostate carcinoma. Scand J Infect Dis 2011;43:64-69.

52. Bae JM, Lee EJ, Guyatt G. Citrus fruits intake and prostate cancer risk: a quantitative systematic review. J Prev Med Public Health 2008;41:159-164.

53. Higgins JPT, Thompson SG. Quantifying heterogeneity in a meta-analysis. Stat Med 2002;21:1539-1558.

54. Wang T, Chang P, Wang L, Yao Q, GUo W, Chen J, et al. The role of human papillomavirus infection in breast cancer. Med Oncol 2012;29:48-55.

55. Effert PJ, Frye RA, Neubauer A, Liu ET, Walther PJ. Human papillomavirus types 16 and 18 are not involved in human prostate carcinogenesis: analysis of archival human prostate cancer specimens by differential polymerase chain reaction. J Urol 1992;147:192-196.

56. Serfling U, Ciancio G, Zhu WY, Leonardi C, Penneys NS. Human papillomavirus and herpes virus DNA are not detected in benign and malignant prostatic tissue using the polymerase chain reaction. J Urol 1992;148:192-194.

57. Sarkar FH, Sakr WA, Li YW, Sreepathi P, Crissman JD. Detection of human papillomavirus (HPV) DNA in human prostatic tissues by polymerase chain reaction (PCR). Prostate 1993;22:171-180.

58. Saad F, Gu K, Jean-Baptiste J, Gauthier J, MesMasson AM. Absence of human papillomavirus sequences in early stage prostate cancer. Can J Urol 1999;6:834-838.

59. Zambrano A, Kalantari M, Simoneau A, Jensen JL, Villarreal LP. Detection of human polyomaviruses and papillomaviruses in prostatic tissue reveals the prostate as a habitat for multiple viral infections. Prostate 2002;53:263-276.

60. Leskinen MJ, Vainionp R, Syrjnen S, Leppilahti M, Marttila T, Kylml T, et al. Herpes simplex virus, cytomegalovirus, and papillomavirus DNA are not found in patients with chronic pelvic pain syndrome undergoing radical prostatectomy for localized prostate cancer. Urology 2003;61:397-401.

61. Bergh J, Marklund I, Gustavsson C, Wiklund F, Grönberg H, Allard A, et al. No link between viral findings in the prostate and subsequent cancer development. Br J Cancer 2007;96:137-139.

62. Balis V, Sourvinos G, Soulitzis N, Giannikaki E, Sofras F, Spandidos DA. Prevalence of BK virus and human papillomavirus in human prostate cancer. Int J Biol Markers 2007;22:245-251.

63. Sfanos KS, Sauvageot J, Fedor HL, Dick JD, De Marzo AM, Isaacs WB. A molecular analysis of prokaryotic and viral DNA sequences in prostate tissue from patients with prostate cancer indicates the presence of multiple and diverse microorganisms. Prostate 2008;68:306-320.

64. Masood S, Rhatigan RM, Powell S, Thompson J, Rodenroth N. Human papillomavirus in prostatic cancer: no evidence found by in situ DNA hybridization. South Med J 1991;84:235-236.

65. Dodd JG, Paraskevas M, McNicol PJ. Detection of human papillomavirus 16 transcription in human prostate tissue. J Urol 1993;149:400-402.

66. Suzuki H, Komiya A, Aida S, Ito H, Yatani R, Shimazaki J. Detection of human papillomavirus DNA and p53 gene mutations in human prostate cancer. Prostate 1996;28:318-324.

67. Wideroff L, Schottenfeld D, Carey TE, Beals T, Fu G, Sakr W, et al. Human papillomavirus DNA in malignant and hyperplastic prostate tissue of black and white males. Prostate 1996;28:117-123.

68. Anderson M, Handley J, Hopwood L, Murant S, Stower M, Maitland NJ. Analysis of prostate tissue DNA for the presence of human papillomavirus by polymerase chain reaction, cloning, and automated sequencing. J Med Virol 1997;52:8-13.

69. Strickler HD, Burk R, Shah K, Viscidi R, Jackson A, Pizza G, et al. A multifaceted study of human papillomavirus and prostate carcinoma. Cancer 1998;82:1118-1125.

70. Gazzaz FS, Mosli HA. Lack of detection of human papillomavirus infection by hybridization test in prostatic biopsies. Saudi Med J 2009;30:633-637.

71. Martinez-Fierro ML, Leach RJ, Gomez-Guerra LS, Garza-Guajardo R, Johnson-Pais T, Beuten J, et al. Identification of viral infections in the prostate and evaluation of their association

with cancer. BMC Cancer 2010;10:326.

72. McNicol PJ, Dodd JG. Detection of human papillomavirus DNA in prostate gland tissue by using the polymerase chain reaction amplification assay. J Clin Microbiol 1990;28:409-412.

73. Chen AC, Waterboer T, Keleher A, Morrison B, Jindal S, McMillan D, et al. Human papillomavirus in benign prostatic hyperplasia and prostatic adenocarcinoma patients. Pathol Oncol Res 2011;17:613-617.

74. Smelov V, van Moorselaar J, Startsev V, Smelova N, Grigorovich E, Meijer C, et al. No high-risk human papillomavirus infection in prostate cancer tissues. Scand J Infect Dis 2011;43:399-400.

75. Tachezy R, Hrbacek J, Heracek J, Salakova M, Smahelova J, Ludvikova V, et al. HPV persistence and its oncogenic role in prostate tumors. J Med Virol 2012;84:1636-1645.

76. Michopoulou V1, Derdas SP, Symvoulakis E, Mourmouras N, Nomikos A, Delakas D, et al. Detection of human papillomavirus (HPV) DNA prevalence and p53 codon 72 (Arg72Pro) polymorphism in prostate cancer in a Greek group of patients. Tumour Biol 2014;35:12765-12773.

77. Yow MA, Tabrizi SN, Severi G, Bolton DM, Pedersen J, Longano A, et al. Detection of infectious organisms in archival prostate cancer tissues. BMC Cancer 2014;14:579.

78. Singh N, Hussain S, Kakkar N, Singh SK, Sobti RC, Bharadwaj M. Implication of high risk Human papillomavirus HR-HPV infection in prostate cancer in Indian population- A pioneering case-control analysis. Sci Rep 2015;5:7822.

79. Roxburgh CS, McMillan DC. Cancer and systemic inflammation: treat the tumour and treat the host. Br J Cancer 2014;110:1409-1412.

80. Chung JI. Prevention of prostate cancer. J Korean Med Assoc 2010;53:135-141. (Korean)

81. Bardia A1, Platz EA, Yegnasubramanian S, De Marzo AM, Nelson WG. Anti-inflammatory drugs, antioxidants, and prostate cancer prevention. Curr Opin Pharmacol 2009;9:419-426.

82. La Vecchia C, Bosetti C. Urological cancer: Aspirin and the risk of prostate cancer mortality. Nat Rev Clin Oncol 2012;9:616-617.

83. Castellsagué X, Schneider A, Kaufmann AM, Bosch FX. HPV vaccination against cervical cancer in women above 25 years of age: key considerations and current perspectives. Gynecol Oncol 2009;115(3 Suppl):S15-S23.

84. Vedham V, Divi RL, Starks VL, Verma M. Multiple infections and cancer: implications in epidemiology. Technol Cancer Res Treat 2014;13:177-194.

85. Sankaranarayanan R, Ramadas K, Qiao YL. Managing the changing burden of cancer in Asia. BMC Med 2014;12:3.

86. Park S, Bae J, Nam BH, Yoo KY. Aetiology of cancer in Asia. Asian Pac J Cancer Prev 2008;9:371-380.

87. Colón-López V, Ortiz AP, Palefsky J. Burden of human papillomavirus infection and related comorbidities in men: implications for research, disease prevention and health promotion among Hispanic men. P R Health Sci J 2010;29:232-240.

88. Konya J, Dillner J. Immunity to oncogenic human papillomaviruses. Adv Cancer Res 2001;82:205-238.

Table 1. The subject articles (n=30) in the adaptive meta-analysis for evaluating Human Papillomavirus 16 Infection as a Potential Risk factor of Prostate Cancer*

Group	Author	YEAR	Ref	Case_T	Case_P	Control_T	Control_P
A	McNicol	1991	38	27	14	61	35
A	Anwar	1992	41	68	11	20	0
A	Ibrahim	1992	40	24	6	29	2
A	Rotola	1992	39	8	6	17	14
A	Tu	1994	42	60	1	1	0
A	Moyret-Lalle	1995	43	17	9	22	7
A	Terris	1997	44	53	2	78	4
A	Noda	1998	45	38	0	71	3
A	Serth	1999	46	47	10	37	1
A	Carozzi	2004	47	26	3	25	0
A	Leiros	2005	48	41	5	30	0
A	May	2008	49	50	10	163	29
A	Silverstre	2009	50	65	2	6	0
A	Aghakhani	2010	51	104	7	104	3
B	Masood	1991	64	20	0	20	0
B	Dodd	1993	65	7	3	10	5
B	Suzuki	1996	66	51	8	51	0

B	Wideroff	1996	67	56	7	42	4
B	Anderson	1997	68	14	0	10	0
B	Strickler	1998	69	63	0	61	0
B	Gazzaz	2009	70	6	0	50	0
B	Martinez-Fierro	2010	71	55	0	75	0
C	McNicol	1990	72	4	4	20	15
C	Chen	2011	73	51	0	11	0
C	Smelov	2011	74	61	2	14	0
C	Tachezy	2012	75	51	1	95	11
C	Whitaker	2013	26	10	0	20	0
C	Michopoulou	2014	76	50	2	30	0
C	Yow	2014	77	115	0	51	0
C	Singh	2015	78	95	30	55	3

* A group: selected articles from Reference [27]; B group: selected articles by comparing subjects between Reference [27] and Reference [25]; C group: selected articles by hand searching ; Ref.: reference number; Case_T: total number of case group; Case_P: positive number of case group; Control_T: total number of control group; Control_P: positive number of control group: OR: odds ratio; L_CI: lower margin of 95% confidence intervals; U_CI: upper margin of 95% confidence intervals

Table 2. Summary odds ratio (sOR) and its 95% confidence intervals (CIs) by meta-analysis with subgroup analysis for evaluating Human Papillomavirus 16 Infection as a Potential Risk factor of Prostate Cancer*

group & subgroup	Number (df)	I^2 statistic (%)	sOR (Fixed) [95% CI]	sOR (Random) [95% CI]
A	14 (13)	27.78	1.669 [1.134, 2.456]	1.574 [0.906, 2.735]
A+B	22 (16)	27.43	1.761 [1.235, 2.509]	1.600 [0.968, 2.643]
A+B+C	30 (21)	37.82	1.851 [1.353, 2.532]	1.719 [1.037, 2.848]
Publication before 2000	16 (12)	34.24	1.772 [1.150, 2.729]	1.565 [0.820, 2.987]
Publication after 2000	14 (8)	47.41	1.946 [1.236, 3.064]	1.961 [0.812, 4.733]

* A group: selected articles from Reference [27]; B group: selected articles by comparing subjects between Reference [27] and Reference [25]; C group: selected articles by hand searching ; df: degree of freedom; Fixed: fixed effect model; Random: random effect model; CI: confidence intervals
